# Vasa vasorum inside out/outside in communication: a potential role in the patency of saphenous vein coronary artery bypass grafts

**DOI:** 10.1007/s12079-018-0483-1

**Published:** 2018-08-04

**Authors:** Andrzej Loesch, Michael R. Dashwood

**Affiliations:** 1 Centre for Rheumatology and Connective Tissue Diseases, Division of Medicine, University College London Medical School, Royal Free Campus, Rowland Hill Street, London, NW3 2PF UK; 2 Division of Surgery and Interventional Science, Faculty of Medical Sciences, University College London Medical School, Royal Free Campus, Rowland Hill Street, London, NW3 2PF UK

**Keywords:** Saphenous vein, Coronary artery bypass, Vasa vasorum, Vascular damage

## Abstract

The saphenous vein (SV) is the most commonly used conduit for revascularization in patients undergoing coronary artery bypass surgery (CABG). The patency rate of this vessel is inferior to the internal thoracic artery (ITA). In the majority of CABG procedures the ITA is removed with its outer pedicle intact whereas the (human) SV (hSV) is harvested with pedicle removed. The vasa vasorum, a microvessel network providing the adventitia and media with oxygen and nutrients, is more pronounced and penetrates deeper towards the lumen in veins than in arteries. When prepared in conventional CABG the vascular trauma caused when removing the hSV pedicle damages the vasa vasorum, a situation affecting transmural flow potentially impacting on graft performance. In patients, where the hSV is harvested with pedicle intact, the vasa vasorum is preserved and transmural blood flow restored at graft insertion and completion of CABG. By maintaining blood supply to the hSV wall, apart from oxygen and nutrients, the vasa vasorum may also transport factors potentially beneficial to graft performance. Studies, using either corrosion casts or India ink, have shown the course of vasa vasorum in animal SV as well as in hSV. In addition, there is some evidence that vasa vasorum of hSV terminate in the vessel lumen based on *ex vivo* perfusion, histological and ultrastructural studies. This review describes the preparation of the hSV as a bypass conduit in CABG and its performance compared with the ITA as well as how and why its patency might be improved by harvesting with minimal trauma in a way that preserves an intact vasa vasorum.

## The saphenous vein as a bypass conduit

**Fig. 1:**
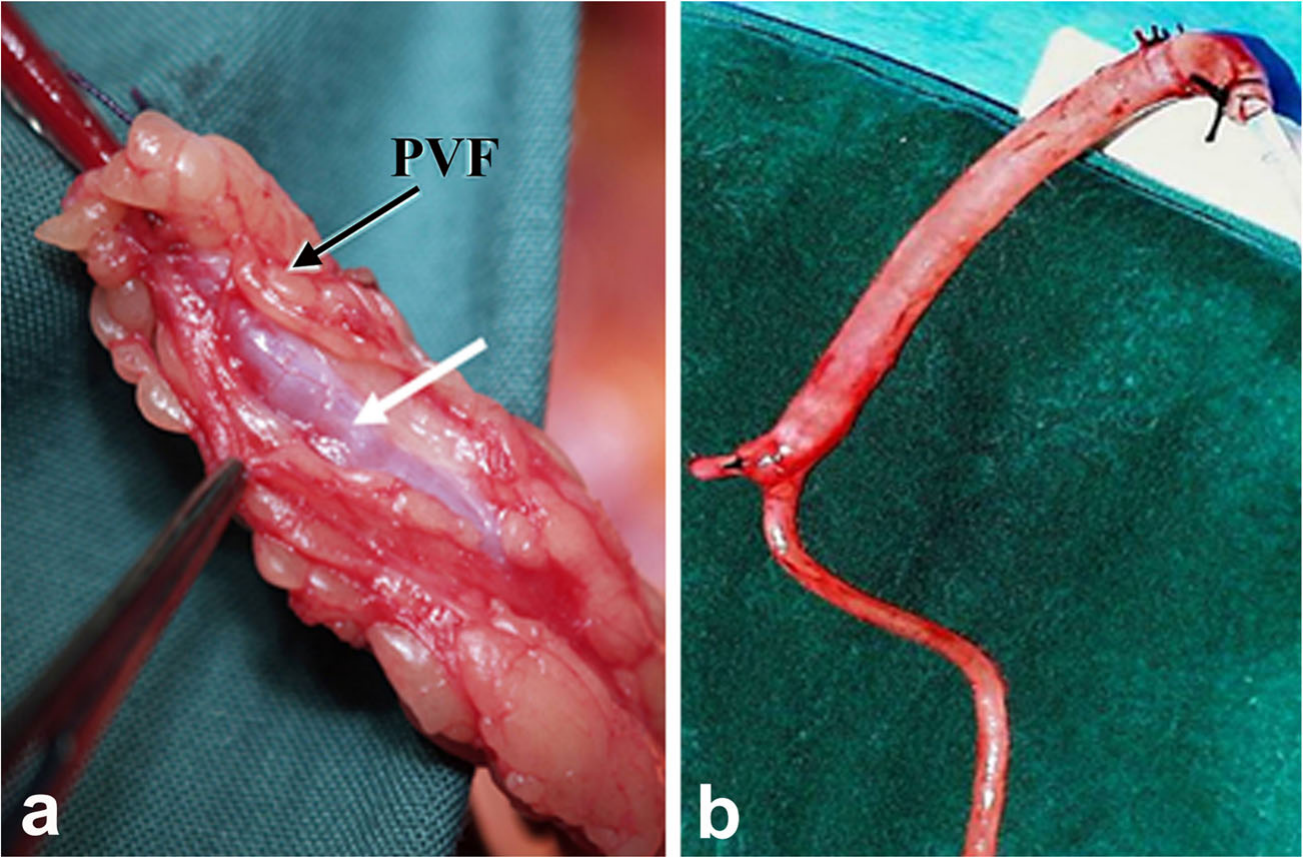
Human SV graft preparations harvested by no-touch (**a**) and conventional (**b**) techniques; white arrow indicate the vein, while the black arrow points to perivascular fat (PVF), which has been removed in (**b**). (Modified from **a** Dashwood et al., Interact Cardiovasc Thorac Surg 2011, 12(2):170–173. doi: 10.1510/icvts.2010.247874 with acknowledgement of Oxford Academic, and **b** from Souza et al., J Thorac Cardiovasc Surg 2006, 132 (2):373–378 with acknowledgement of Elsevier)

In a recent issue of Nature Reviews in Cardiology de Vries and colleagues discuss the use of the saphenous vein (SV) as a conduit for surgical revascularization in patients undergoing coronary artery bypass graft surgery (CABG) (de Vries et al. 2016). In their review the authors comment on the high failure rate of human SV (hSV) grafts, studies into underlying causes of graft failure and recent therapeutic options aimed at improving graft patency. The hSV is the most commonly used conduit for CABG with the other main conduits being the internal thoracic artery (ITA) and radial artery (RA). The hSV is the conduit of choice since it is expendable, as deeper vessels maintain blood flow to superficial tissues after its removal, the extensive length (potentially 60 cm, ~30 cm from each leg) of this vein allows for multiple grafts and its superficial position renders it easily accessible (Tsui and Dashwood 2002). A recent Perspective in the New England Journal of Medicine, (Jones 2017) highlights the important contribution of the Argentinian cardiac surgeon, Rene Favaloro, who introduced the hSV as a conduit for coronary revascularization in 1968 (Favaloro 1968). In general, both the arterial grafts are harvested with their outer pedicle of tissue intact whereas, according to Favaloro’s original instructions, “Care must be taken to dissect only the vein, avoiding as much as possible the adventitia that surrounds it” (Favaloro 1969). This is now the most commonly used, ‘conventional’ (CT), method employed by the majority of cardiac surgeons worldwide and, when harvesting the hSV this way, the vessel’s outer pedicle of fat is removed. In addition, the adventitia is stripped or damaged causing trauma that most likely affects both early- and long-term graft patency. An atraumatic, no-touch (NT), method of harvesting the hSV was introduced over two decades ago where the hSV is removed complete with its surrounding cushion of fat intact and the adventitia intact and undamaged (Souza 1996). Here, Fig. 1 demonstrates examples of NT and CT graft preparations of hSV for CABG. No touch hSV grafts (NTSVGs) have an improved patency over CT hSV and are comparable to the ITA at up to 16 years follow up (Samano et al. 2015). A major factor suggested to explain this observation is that NTSVG preparations do not go into spasm since there is no direct handling of the hSV by surgical instruments, high pressure intralumenal saline distension is not required and endothelial and vascular smooth muscle cell (VSMC) damage is reduced (Souza 1996; Souza et al. 1999; Tsui et al. 2001; Ahmed et al. 2004; Vasilakis et al. 2004; Verma et al. 2014). Preservation of an intact endothelium and endothelium-derived nitric oxide (NO) is suggested to account for abolition of spasm at harvesting, reduced platelet aggregation/thrombus formation (early graft occlusion) and reduced neointimal hyperplasia (mid-term occlusion) (Tsui et al. 2001, 2002). The minimised surgical trauma preserves the shape and phenotype of VSMCs thus impacting on proliferation, neointimal hyperplasia and atheroma formation (Ahmed et al. 2004; Verma et al. 2014).

## Saphenous vein vasa vasorum

An interesting observation made at completion of NTSVG insertion and proximal graft anastomosis is the retrograde filling of the adventitial vasa vasorum at removal of vascular clamps (Souza et al. 1999). This immediate restoration of blood supply has been suggested to restore/preserve transmural flow, contributing to the maintenance of a ‘healthy’ graft (Dashwood and Tsui 2013). Also, since the explanted hSV graft is disconnected at both distal and proximal ends, filling of the vasa vasorum is most likely via terminations in the vein lumen and tributaries. Indeed, there are studies in support of such an arrangement of vasa terminations in parental vein lumen. For example, luminal vasa vasorum have been demonstrated in experimental vein grafts using silicone rubber casts where luminally originating vasa vasorum in the neointima were often visible along the suture line of the anastomosis and distributed throughout the media and adventitia and connecting to the original vasa (Ohta and Kusaba 1997). These observations support earlier evidence of retrograde filling of vasa vasorum terminating within the vessel lumen (Corson et al. 1985; Crotty 1989, 2003). A connection between the vasa vasorum and lumen has been demonstrated in canine vein grafts where, *in vivo*, (luminal) endothelial integrity is maintained even in the absence of intraluminal blood flow (Corson et al. 1985). In these studies it was shown that, using intravenous fluorescein, blood flow through the vasa vasorum supplies oxygen and nutrients to the entire vein wall including the endothelium. These results suggest that the luminal endothelium is supplied by nutrients directly via the vasa vasorum rather than by diffusing through the deepest layers of the media as previously stated by Brock (1977).

Classically, as oxygenated blood passes through the lumen of arteries, the endothelium and VSMCs receive sufficient oxygen and nutrients by diffusion thus, the role of the vasa vasorum is less significant. However, in veins, the vasa vasorum represents a microvessel network in the vessel wall where its principle role is to supply blood to the wall as luminal oxygen and nutrient levels are low. Hence, the vasa vasorum of veins penetrate closer towards the intima than those of arteries and “are seen to advantage in the thick walls of the saphenous vein” (Ham 1974). The importance of the vasa vasorum in maintaining ‘healthy vessels’ is often overlooked and these microvessels are damaged in various forms of vascular surgery, in particular bypass procedures for revascularization. The subsequent interruption of transmural flow renders the vessel wall ischaemic, a condition initiating many processes involved in graft occlusion. In experimental animal models it has been shown that occlusion of the vasa vasorum by a close fitting external collar (Barker et al. 1992, 1993), or adventitial removal (Barker et al. 1994), results in neointimal hyperplasia and atherosclerosis, both characteristics associated with mid- and long-term vein graft failure.

## Luminal termination of vasa vasorum?

**Fig. 2:**
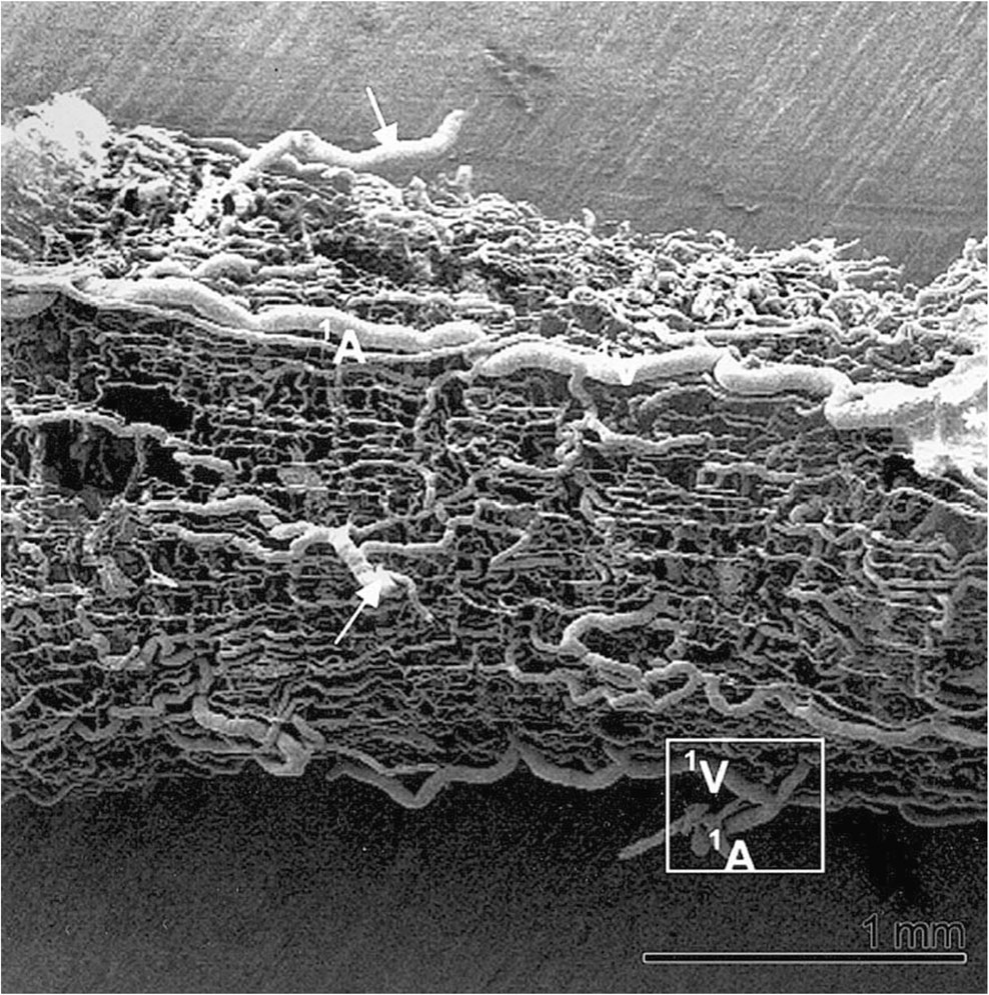
Vasa vasorum of hSV observed by scanning electron microscopy. Note vascular corrosion cast of a spare segment of the proximal portion of the hSV for CABG revealing the complex arrangement of vasa vasorum from an adventitial view; arrows mark the feeding and/or draining vessels infiltrating the SV every 0.5–1.5 cm. A = artery, V = vein. (Reproduced from Lametschwandtner et al., Anat Rec 2004, Part A 281A:1372–1382 [Wiley-Liss, Inc.], which we gratefully acknowledge)

Although the above mentioned studies provide some evidence for vasa vasorum terminating in the vein lumen many, if not the majority, of studies tend to disagree. This is clear from the previously cited publication by Brock (1977), who maintains the depth of penetration of the vasa vasorum through the media extends to within “36 μm of the endothelial layer [i.e. the lumen] in a human long saphenous vein”, in support of earlier, similar, statements (Wyatt et al. 1964). A number of ‘tracing’ studies have been reported using either India ink or vascular corrosion casts to determine the 3 dimensional complex structure of the vasa vasorum in either varicose hSVs (Kachlik et al. 2008) or hSVs obtained from patients undergoing aortocoronary bypassing (Kachlik et al. 2007). Here, Fig. 2 demonstrates a representative example of vasa vasorum in hSV observed after corrosion of vascular casts. Certainly, the 3 dimensional distribution of vasa vasorum that are revealed using scanning electron microscopy of resin casts are most striking and provide the opportunity to illustrate the course of vasa vasorum within the vessel wall as well as to calculate certain morphometric variables such as vasa diameters, branching distances and angles (Lametschwandter et al. 2004; Kachlik et al. 2007, 2008). In these studies, images generated using India ink are less striking and less suitable for quantitative assessment. The vascular bed of the lower extremities of cadavers was also used that were injected 12–24 h post mortem with India ink via the external iliac arteries using manual pressure. The corrosion casting was performed on segments of the middle or proximal hSV from patients undergoing CABG. In all cases hSV segments for CABG were taken with a thick layer of surrounding perivascular adipose tissue, whereby great care was taken to avoid any direct mechanical contact with the vein to prevent artificial venous constriction (essentially using the NTSVG harvesting technique described by Souza 1996). Specimens (without any intraluminal washing and use of vasodilators) were stored in ice-cooled heparinized saline and cast within 6–10 h after harvesting and then examined under scanning electron microscopy. Whilst the examples provided of corrosion casts are impressive, Kachlik et al. (2007) comment that there are likely to be a number of issues affecting the flow of the resin due to its viscosity, which is around 5–10 times higher than that of blood. Also, one should consider the effects of processing and/or post-mortem delay as well as possible tissue shrinkage. The time- and post-mortem-associated factors may limit the access and flow of the tracers used and provide an inadequate impression of the network of the vasa vasorum in hSV used for revascularization in CABG patients. A very recent study from this group used standard histological techniques to assess the distribution of vasa vasorum in walls of failed aorto-coronary venous grafts (Stingl et al. 2018). Here, time-related qualitative evaluation was performed on diseased hSV grafts in an attempt to assess the extent of (neo)vascularization related to graft pathology and re-grafting. While proliferation of vasa vasorum from the adventitia to the outer layers of the media were observed between 7 and 24 months after implantation, proliferation throughout the entire atherosclerotic media and hyperplastic intima continued for a much longer time interval. These observations therefore indirectly agree with the well-established data that vasa vasorum play a central role in the pathogenesis of atherosclerosis (Gingras et al. 2009; Mulligan-Kehoe and Simons 2014; Boyle et al. 2017; Sedding et al. 2018). Interestingly, Stingl et al. (2018) were unable to identify luminal terminations of vasa vasorum in the hSV samples studied. This is not surprising since most were pathological samples of failed grafts with varying degrees of neointimal hyperplasia or atherosclerosis. Furthermore, from the appearance of the examples provided the hSV grafts had been harvested with perivascular fat (PVF)/perivascular adipose tissue (PVAT) removed using the CT, a situation that causes significant histological and immunohistochemical damage to the vasa vasorum (Ahmed et al. 2004; Dreifaldt et al. 2011, 2013).

**Fig. 3:**
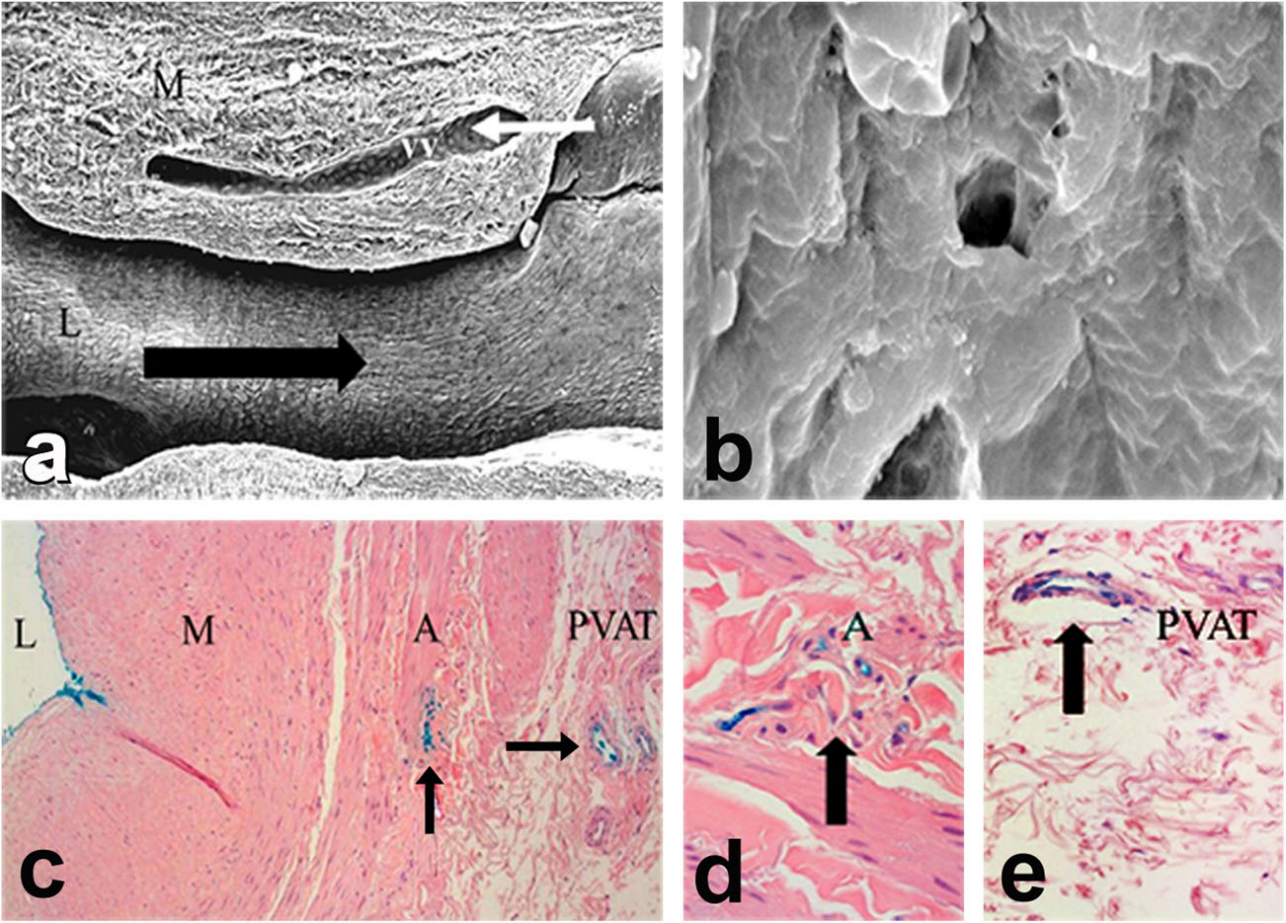
Possible luminal termination of vasa vasorum in NTSVG preparations as examined with the scanning electron microscope **(a, b)** and light microscope **(c-e)**. **a** Note vasa vasorum (vv) in sections of vein media (M). The venous ends (white arrow) of these vessels drained directly into the lumen (L); the black arrow points the direction of blood flow. Original magnification ×70. (Modified from Souza et al., Scand J Cardiovasc Surg 1999, 33(6):323–329 with acknowledgement of Scandinavian University Press). **b** Showing a potential opening (4–5 μm) of vasa vasorum within the vein luminal endothelium. Original magnification ×3000. (Modified from Dreifaldt et al., J Thorac Cardiovasc Surg 2011, 141(1):145–150 with acknowledgements of Elsevier). **c-e** Transverse sections of NTSVG preparations perfused with Indian ink (*blue*) show staining (arrows) at the lumen (L) as well as at vasa vasorum within the adventitia (A) and perivascular adipose tissue (PVAT); M = media. Original magnifications (**c**) ×95, (**d, e**) ×190. (**c-e** Modified from Fernández-Alfonso et al., Curr Vasc Pharmacol 2016, 14(4):308–312 with acknowledgement of Dr. M. Dreifaldt and Bentham Science Publishers Ltd)

**Fig. 4:**
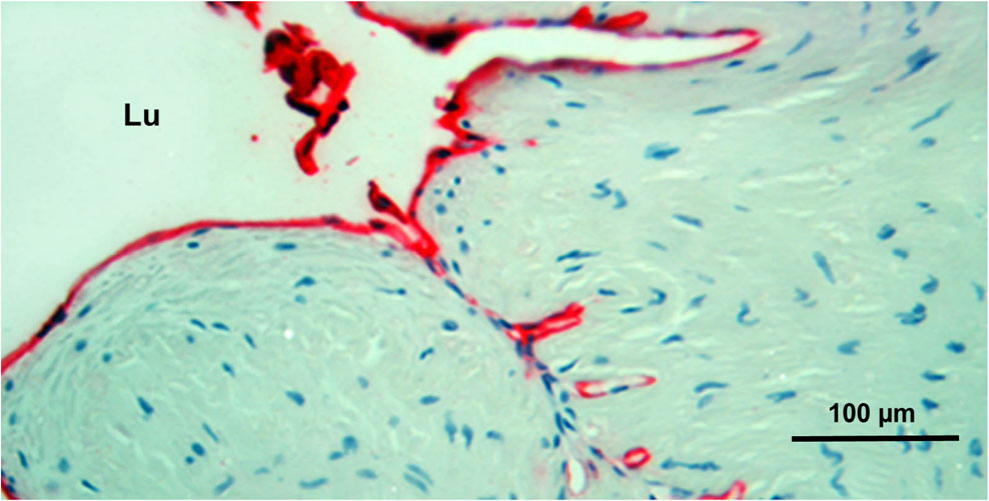
Vasa vasorum in close proximity to the lumen of NTSVG. Note red CD31-endothelial immunostaining concentrated close to the NTSVG lumenal folds as seen in a circumferential section of the vein; Lu = lumen. (Modified from Dashwood et al., Angiology 2004, 55(4):385–395 with acknowledgement of SAGE Publishing, Westminster Publications, Inc., USA)

A recent study using India ink provides supporting evidence for the luminal termination of vasa vasorum in the hSV where this microvessel network extends from the vessel lumen, courses through the media and adventitia to the capillaries within the vein’s surrounding cushion of PVAT. Here, segments of hSV from patients undergoing CABG were perfused under normal cardiovascular conditions with India ink via a cannula inserted in the lumen. Histological examination of sections taken from these veins showed staining of the luminal endothelium and the vasa vasorum throughout the vessel wall and extending to the capillary network within the PVAT (Fernández-Alfonso et al. 2016). Figure 3 demonstrates a possible direct link between vasa vasorum and the lumen of hSV harvested for CABG, while Fig. 4 shows the “concentration” of vasa at the region of hSV intimal folds that may reach the lumen.

In the light of the above data, the role of preserved/intact vasa vasorum in hNTSVGs in CABG patients should be recognised as “beneficial” since physiological communication between intima, media, adventitia and PVAT remains uninterrupted in such conditions (more on this subject in the “Vasa vasorum: vein versus artery” section below). However, it is important to stress that at present little is known regarding the luminal termination of vasa in hSV used for CABG. The most compelling evidence has been previously described in canine SV, where it is known that such terminations of vasa at the SV lumen can be opened by a complex structure – the agger, which is sensitive to changes in blood turbulence and conditions affecting flow through vasa microvessels (see Crotty 2011).

## Vasa vasorum *in vivo*: contribution to saphenous vein graft performance

**Fig. 5:**
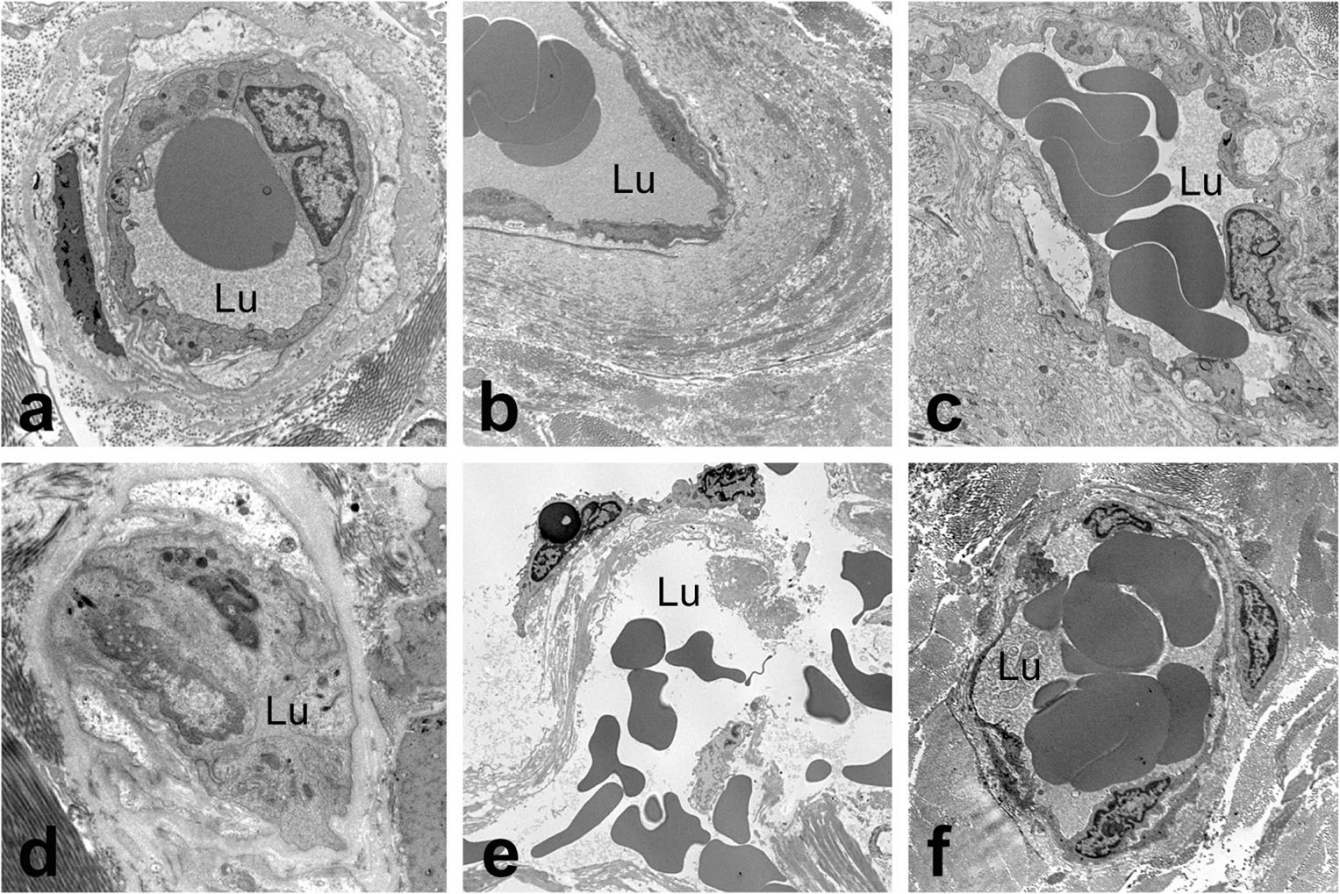
Transmission electron microscopy of diverse features of vasa vasorum in NTSV **(a-c)** and CTSV **(d-e)** graft preparations. Note the open lumen in all vasa vessels in NTSV preparations (**a-c**); also note the presence of red blood cells within the luminal space *(Lu).* In CTSV preparations vasa are frequently collapsed or constricted so that the luminal space is closed and not readily visible (**d**). In **e** note the damage to a vasa vessel by CT harvesting; also note red blood cells outside the vascular pool. In contrast, image **f** shows a rather well-preserved vessel. Original magnifications: **a, d** × 5600; **b, e, f** × 2650; **c**, ×4400. (Modified from Dreifaldt et al., J Thorac Cardiovasc Surg 2011, 141(1): 145–150 doi: 10.1016/j.jtcvs.2010.02.005, with acknowledgement of Elsevier)

**Fig. 6:**
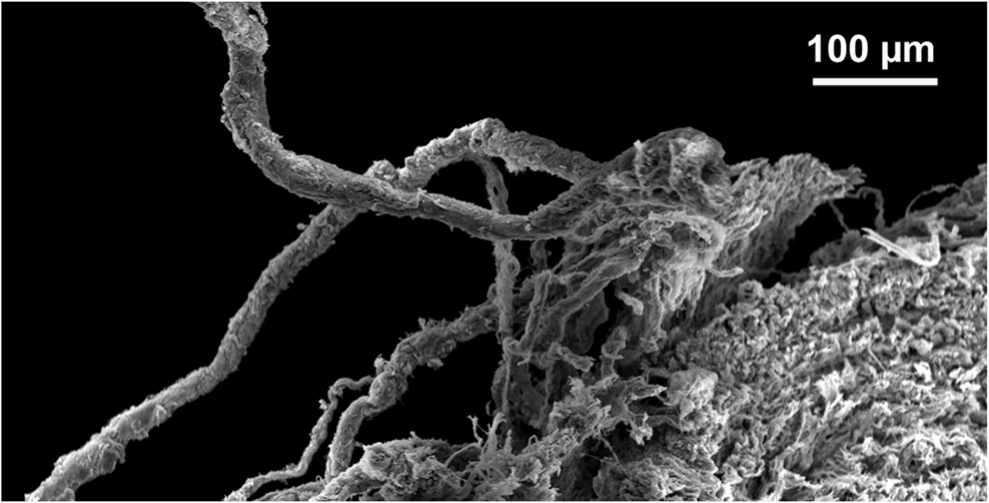
Scanning electron microscopy of conventionally harvested SV. Note that the vasa vasorum vessels together with a part of adventitia are partially detached from the vein wall and exposed to the external environment. (Reproduced from Vasilakis et al., Vasc Dis Prev 2004, 1(2):133–139, with acknowledgement of Bentham Science Publishers Ltd)

**Fig. 7:**
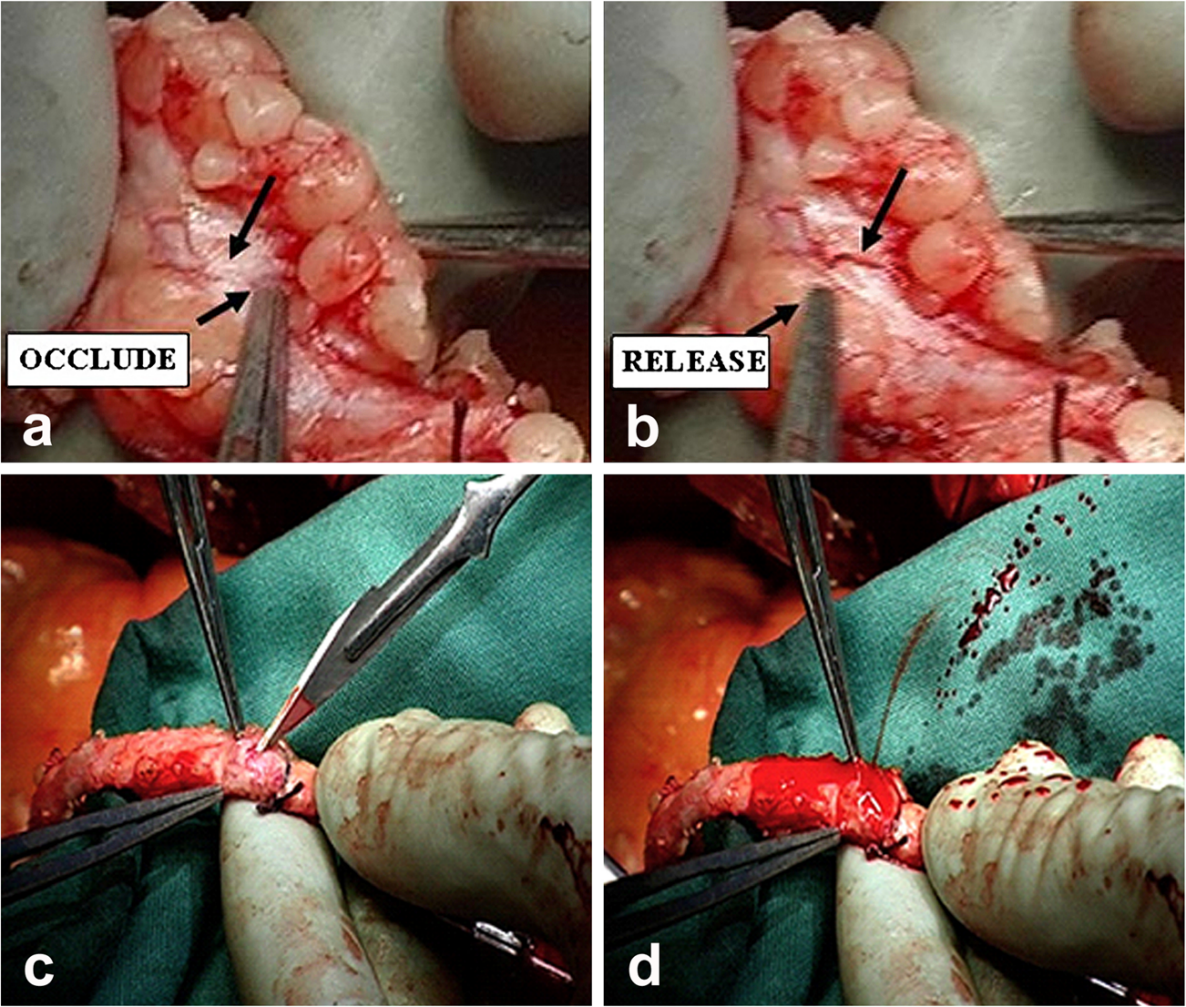
Retrograde filling of NTSVG adventitial vasa vasorum during CABG – perfusion of blood from cardiopulmonary bypass via the lumen. **a** A vasa vessel is not visible (in the area indicated by arrows) due to the occlusion by surgical forceps, while in the **b**, the vasa vessel is clearly seen when the occlusion is released and blood flow is restored. **c-d** Bleeding from an incised adventitial vasa vasorum. (Both screen shots from the video in Dreifaldt et al., J Thorac Cardiovasc Surg 2011, 141(1): 145–150, with acknowledgements of Elsevier)

When harvesting the hSV conventionally for CABG, as described by Favaloro (1969), the surrounding cushion of fat is removed, the adventitia partially stripped and the SV distended at high pressure to overcome the venospasm that often occurs in response to surgical trauma. These procedures cause tissue and/or cell damage to all vessel layers, damage that affects graft patency (Ahmed et al. 2004; Vasilakis et al. 2004; Dashwood and Tsui 2013; Verma et al. 2014). In particular, regarding vascular damage, there is evidence, not only of structural alterations to endothelial cells and VSMCs, but also the constriction/collapse of some vasa vasorum and ‘clumping’ of erythrocytes (Ahmed et al. 2004; Dreifaldt et al. 2011); Here, Fig. 5 demonstrates the above-mentioned features of vasa vasorum in CT hSV that also display the presence of extravasated red cells (Fig. 5e) – a feature that may contribute to vascular pathology (Nagy et al. 2010). Damage to the vasa vasorum of conventionally harvested hSV is also observed using scanning electron microscopy (Fig. 6) where ‘disconnection’ of adventitial vessels/microvessels is evident (Vasilakis et al. 2004). By contrast, where NTSVGs are removed atraumatically and with minimal vascular damage, the vasa vasorum remain intact with normal endothelial cell morphology and VSMCs also maintaining a normal appearance (Ahmed et al. 2004). Also, when using scanning electron microscopy, small openings were observed within the luminal endothelial lining of the hSV (Fig. 3), openings taken to represent possible terminations of vasa vasorum, presumably extensions of the adventitial-medial microvessel network (Souza et al. 1999; Dreifaldt et al. 2011). These features are likely to have functional relevance since the filling of adventitial vasa vasorum has been described in NTSVGs perfused with blood from the arterial line of the cardiopulmonary bypass machine before completion of the proximal anastomoses, or at completion of proximal anastomosis and removal of vascular clamps during CABG (Souza et al. 2009; Dreifaldt et al. 2011). Here, Fig. 7 demonstrates retrograde filling of NTSVG adventitial vasa vasorum during CABG. Most importantly, data presented above suggests that, at completion of graft insertion, retrograde (coronary) arterial blood flow occurs from the graft lumen via the medial to the adventitial vasa vasorum, a situation that has been confirmed *ex vivo* on isolated segments of NTSVG perfused with blood from the cardiopulmonary bypass machine (Souza et al. 2009; Dreifaldt et al. 2011).

## Vasa vasorum: vein versus artery

**Fig. 8:**
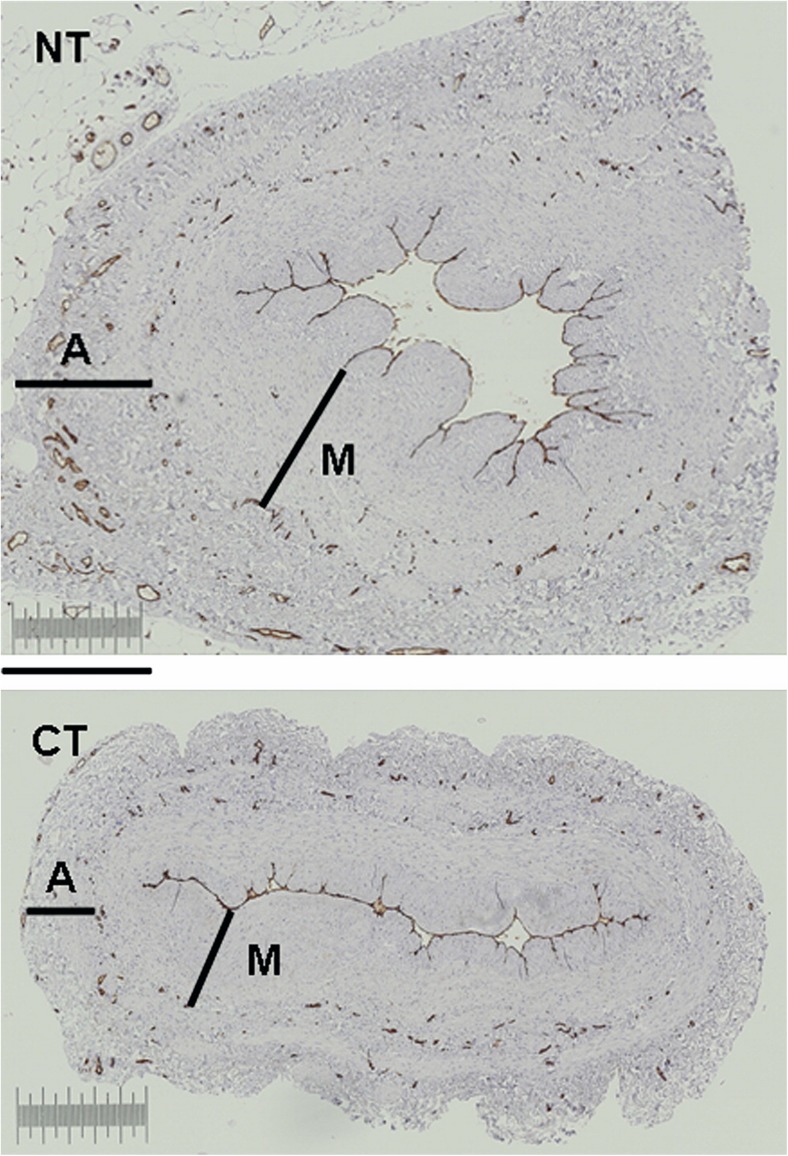
Quantification of vasa vasorum. Representative transverse sections of no-touch (NT) and conventionally harvested (CT) hSV segments from a CABG patient used for quantification of CD34-immunolabeling (*brown* stain) of endothelial cells identifying the vein’s vasa vasorum; A = Adventitia; M = media. Bar = 0.5 mm. (Modified from Dreifaldt et al., J Thorac Cardiovasc Surg 2011, 141(1):145–150, with acknowledgement of Elsevier)

**Fig. 9:**
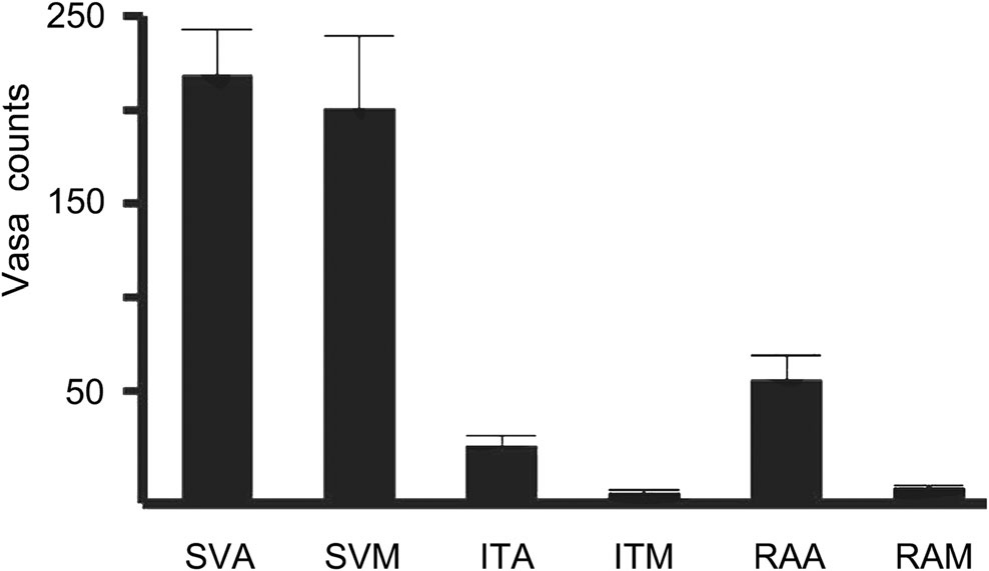
Quantitative assessment comparing vasa vasorum density in bypass conduits from patients undergoing CABG surgery. Note dominant vasa vasorum density in SV adventitia (SVA) and SV media (SVM) as compared to internal thoracic artery adventitia (ITA) and media (ITM), as well as radial artery adventitia (RAA) and media (RAM). (Adapted from Dreifaldt et al., Angiology 2013, 64(4):293–9. doi: 10.1177/0003319712443729. Epub 2012 May 7, with acknowledgement of SAGE Publishing, Westminster Publications, Inc., USA)

It is well recognised that vasa vasorum of veins are more pronounced than in arteries where oxygen and nutrients are supplied by the luminal (arterial) blood. The vasa vasorum of veins are classically described as being situated within the adventitia and the media (Ham 1974) with some suggesting these microvessels penetrate deep within the media and in close proximity to the intima/lumen (Wyatt et al. 1964; Brock 1977). The study described by Dreifaldt et al. (2011) provides evidence for the luminal termination of vasa vasorum in the hSV. Also, in this study the density, size and total area of the vasa vasorum in the media and adventitia of transverse histological sections was assessed by computer-assisted morphometry comparing NTSVG with CT hSV grafts (Fig. 8). While there was no significant difference between NTSVG and CT hSV grafts regarding the density or size in these vessel layers, there was a significantly greater total area in both the media and adventitia of NTSVG compared with CT SV. A similar, follow up, study was performed where the number of vasa vasorum in the media and adventitia of NTSVG sections were compared with the media and adventitia of the ITA and RA. Here, it was confirmed that the number of vasa vasorum in the media and adventitia of NTSVGs was similar. However, the number of vasa vasorum in these layers of NTSVGs was significantly higher than of the ITA and RA (Dreifaldt et al. 2013); see Fig. 9. In addition, this study provides quantitative support for the previously described ‘penetration’ of vasa vasorum in veins versus arteries (Ham 1974) where the number of vasa in the media were significantly lower than in the adventitia of both the ITA and RA (Dreifaldt et al. 2013). These results suggest that the vasa vasorum are more important in maintaining transmural blood supply in veins than in arteries and that, unlike arteries, this high density microvascular network is sustained in the media and approaches the lumen. Therefore it seems reasonable to assume that preservation of an intact vasa vasorum in hSV for CABG plays an important role in maintaining a healthy vessel and consequently in improved graft performance. Conversely, the damage to the vasa vasorum that occurs using CT harvesting for CABG plays a significant role in the inferior performance of the hSV when compared to the ITA.

The study by Loop et al. (1986) showed the ITA to have significantly better patency than the hSV at 10 years (86% versus 71% respectively). However, this data was obtained on patients receiving CT hSV grafts. Subsequently, follow up studies on patients receiving NTSVG and ITA grafts show that NTSVG patency is superior to hSV harvested conventionally at up to 16 years, with a patency comparable to the ITA (Samano et al. 2015). Although other aspects of NTSVG, such as preservation of PVAT (Dashwood et al. 2007, 2009, 2011) and reducing damage to the luminal endothelium (Souza et al. 1999; Tsui et al. 2001, 2002) are important, hSV graft patency is also improved by keeping the vasa vasorum intact and maintaining transmural blood flow.

The advantages of the NTSVG technique is becoming recognised and the technique adopted by an increasing number of cardiac centres worldwide. Indeed, the recent review by deVries and colleagues (deVries et al. 2016) states “Therefore, preservation of vein graft patency is essential for long-term surgical success. With the exception of ‘no-touch’ techniques and lipid-lowering and antiplatelet (aspirin) therapy, no intervention has hitherto unequivocally proven to be clinically effective in preventing vein graft failure”. Noteworthy, here, is the popularity of minimally invasive, endoscopic vein harvesting (EVH) of the hSV for CABG in the USA where the majority of hSV are prepared by this procedure (Dacey et al. 2011). EVH was introduced more than two decades ago (Lumsden et al. 1996) as a technique that reduces leg wound complications, infection rate and pain and improves cosmesis. Despite these benefits of EVH there is disagreement regarding the patency of hSVs prepared by CT versus EVH where, at best, patency of EVH grafts are ‘comparable’ to CT grafts (Perrault et al. 2004; Yun et al. 2005). There are also conflicting reports comparing EVH to CT harvesting on SV morphology. Some suggest there are no detrimental effects on vein morphology, endothelial structure, or function (Fabricius et al. 2000; Griffith et al. 2000; Black et al. 2001), that there is better preservation (Nowicki et al. 2004) or no difference (Griffith et al. 2000; Meyer et al. 2000) in endothelial integrity between EVH and CT hSV grafts. While the majority of these studies focus on damage to the endothelium, there are a few histological examples showing marked damage to the adventitia of EVH hSV grafts (Nowicki et al. 2004; Kiani and Poston 2011), a factor that seems to have been previously overlooked. As with CT harvesting, the surgical trauma caused using EVH, particularly to the outermost vessel layers, is most likely to damage the vasa vasorum and have a deleterious effect on performance of hSV used as grafts in CABG (for review see Kopjar and Dashwood 2016). The physiological importance of vasa vasorum has recently been highlighted by Boyle et al. (2017), who state, “There is ample evidence that dysfunctional vasa vasorum are directly involved in all stages of atherosclerosis development, making them prime candidates for therapeutic intervention”.

**Fig. 10:**
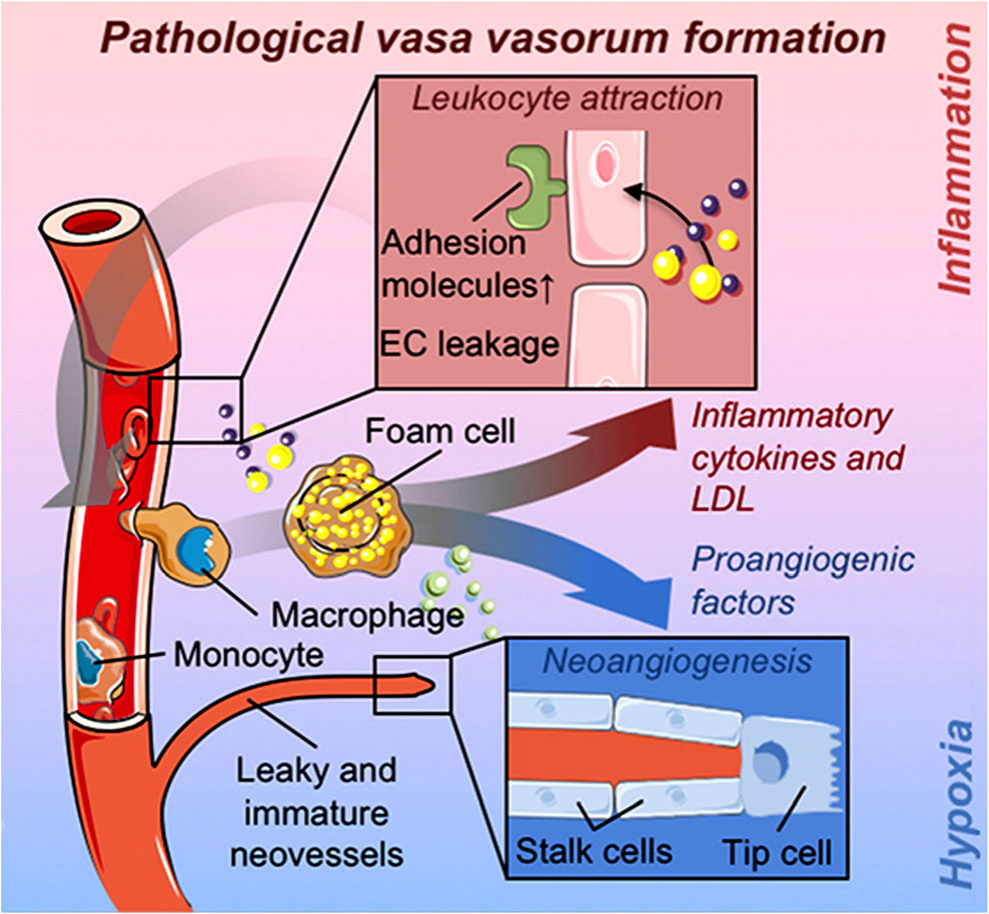
A highlight of pathological formation of adventitial vasa vasorum. Note the events involving, among others, adhesion molecules, leukocytes, inflammatory cells, cytokines, low-density lipoproteins (LDL) or pro-angiogenic factors participating in neo-angiogenesis, leading to eventual projection of neovessels to the media and subintima. (A fragment of Fig. 1 from Sedding et al. (2018), Front Immunol Apr 17;9:706. doi: 10.3389/fimmu.2018.00706. ECollection 2018, which we gratefully acknowledge)

Indeed, several studies show that dysfunction of vasa may stimulate a cascade of pathological processes involving molecular signalling leading in essence to atherosclerosis and vascular disease (for review see Sedding et al. 2018); see Fig. 10. Such a situation may arise during CT harvesting of hSV where, when ‘stripping’ the adventitia, damage and a substantial reduction in vasa occurs (Dreifaldt et al. 2011). This reduction in vasa may eventually lead to re-microvascularization of hSV CT graft adventitia (or rather its remnant) in parallel with the increase of negative inflammatory processes leading to atherosclerosis. Therefore, as Sedding et al. (2018) indicate, there is a growing interest in the understanding of the role and consequences of microvascular expansion in the vessel wall in both physiological and pathophysiological circumstances (also see Gingras et al. 2009; Mulligan-Kehoe 2010; Mulligan-Kehoe and Simons 2014; Boyle et al. 2017). It seems reasonable to assume that the disturbance/pathology of a functioning vasa vasorum also applies to hSV conduits used for CABG, where damage (at times severe) to the vasa microvascular system is inflicted during CT harvesting. Therefore there is a high risk of developing hypoxic or ischaemic conditions in the hCTSV graft wall (at least in the early stages after grafting) and initiating “harmful” processes that affect graft patency. Indeed, the previously mentioned study of failed explanted aorto-coronary hCTSV grafts showed that angiogenesis occurs after the graft was implanted to the coronary bed (Stingl et al. 2018). Interestingly, increased linear proliferation of vasa vasorum into the media and scattered within the intima can be observed in the walls of thrombophlebitic hSVs likely to be influenced by altered venous pressure and lack of oxygen (Chu et al. 2013). In relation to improving the outcome of hSV grafts in CABG patients it seems more appropriate to use undamaged hNTSVGs (Souza 1996), in preference to damaged hCTSVGs; the latter frequently requires physical intervention e.g. mechanical support using external stents (Murphy et al. 2007) or pharmacological intervention targeting vascular growth factors (Pages and Pouyssegur 2005; Sedding et al. 2018).

In the recent article published in the New England Journal of Medicine the RADIAL investigators, an international group of cardiologists and cardiac surgeons, reported a patient-level combined analysis of randomized, controlled trials to compare RA and hSV grafts for CABG in over 1000 patients. The results of this analysis showed there to be an inferior patency of hSV coronary grafts compared to RA grafts as well as a nominally lower incidence of myocardial infarction and a lower incidence of repeat revascularization (Gaudino et al. 2018). However, in this study the details of graft harvesting procedure were not discussed in any detail and we presume that hSV grafts were routinely harvested by CT and therefore likely to have been stripped and distended at the time of CABG surgery. While the mean follow-up time in the RADIAL investigator study was 50 ± 30 months it is noteworthy that the long-term patency of hSVs were superior to the RA (Dreifaldt et al. 2011) and comparable with ITA grafts (Samano et al. 2015) over a longer follow up period when NTSVGs were used. As vascular researchers we believe there is a worrying trend whereby minimal details of harvesting procedure are provided in many cases with no apparent consideration regarding the effects of vascular damage on graft performance. Indeed, comparing CT with NTSVGs may be said to be like “mixing apples with oranges”, a term recently used to criticise the inclusion criteria of certain meta-analyses (Milojevic et al. 2018). While the important role of the endothelium and endothelium-derived factors in graft patency is well-established, ‘mechanical’ damage, due to handling by surgical instruments and/or traction (eg when using EVH) as well as by distension (Souza 1996; Ahmed et al. 2004), to other vascular structures is often overlooked. When hSV are harvested by CT the vasa vasorum is severely damaged, damage likely to initiate the processes of atherosclerosis and graft occlusion associated with the poor performance of this conduit, particularly when compared with the superior patency of the ITA. If hSVs are prepared as NTSVGs normal vessel structure is maintained, a functioning vasa vasorum is preserved and transmural blood supply restored. Consequently, oxygenated arterial blood may be retrogradely delivered from the lumen of the graft where the vasa may play a protective role in protecting the graft wall against ischemia (Souza et al. 2006; Dreifaldt et al. 2011). Furthermore, an intact vasa vasorum may potentially affect graft performance by allowing bidirectional delivery of cell- or tissue-derived signalling molecules throughout the vessel wall.

## Vasa vasorum and hypoxia

Throughout this review we stress the importance of the vasa vasorum in relation to the preservation of the oxygen supply to the wall of SV grafts and the maintenance of communication between intimal, medial and adventitial components/cells via blood flow in this microvascular network. The involvement of the vasa vasorum in normal versus pathological blood vessels has mainly focussed on arteries, particularly in conditions where blood flow is reduced due to narrowing of the lumen caused by intimal hyperplasia or atherosclerosis. In their review on this subject, Ferns and Heikal (2017) discuss the anoxemia theory where a key factor in the development of atherosclerosis is suggested to be due to an imbalance between the demand and supply of oxygen in the arterial wall. In particular the authors mention hypoxia-inducible factor (HIF-1) 1α and its important role as ‘a master regulator of oxygen homeostasis’. Apart from being associated with VSMCs and inflammatory regions of atherosclerotic plaque HIF-1 is also located to perivascular tissues where it regulates certain genes involved in vascular function including vascular endothelial growth factor (VEGF), nitric oxide synthase (NOS) and endothelin. Thirty decades ago elegant ‘cinematographic’ studies on post-mortem atherosclerotic coronary arteries revealed microvessels growing from native vasa vasorum and extending from the adventitia to the thickened intima of atherosclerotic vessels (Barger et al. 1984; Kamat et al. 1987). In more advanced lesions such neovascularization may be seen as an important, beneficial, process in restoring blood flow, and therefore oxygen supply, to the vessel wall. Such new vessel growth/ neoangiogensis is now known to occur in response to physical stimuli such as shear stress and hypoxia involving angiogenic growth factors that are regulated by HIF-1 include angiopoietin 2 (ANGPT-2), placental growth factor (PLGF), stem cell factor (SCF), stromal cell-derived factor 1 (SDF-1), and platelet-derived growth factor B (PDGF-B) (Semenza 2010). Also, under hypoxic conditions there is increased synthesis of heparan sulphate in microvascular endothelial cells which has been shown to stabilize the vasa vasorum and to promote endothelial cell growth (Mollmark et al. 2012) via fibroblast growth factor 2 (FGF-2) receptors (Li et al. 2002).

Whereas the majority of studies relating to the effect of hypoxia in vessel pathology have been mostly on arteries, some have also included hSV material. The importance of maintaining blood and oxygen supply to all layers of the vessel wall has recently been supported by an elegant *ex vivo* culture system using hSV (Piola et al. 2016). This culture system, employing excess hSV material from patients undergoing CABG, provides the ability to generate an oxygen gradient between the luminal and adventitial sides, thereby mirroring the conditions observed in healthy, intact hSV. There is strong evidence that adventitial hypoxia (that may occur in CT hSV grafts) stimulates the process of *neo*-vascularization of the vasa vasorum, which subsequently predisposes arterialized hSV conduits to restenosis (Cox et al. 1991; Piola et al. 2016). We might therefore consider the possibility that varying oxygen concentrations may have different effects on the luminal hSV endothelium than on the ‘microvascular endothelium’ of the vasa vasorum. For example, it is well established that the vasa vasorum of intact hSV is supplied by both arterial and venous blood (Kachlik et al. 2003, 2007; Lametschwandtner et al. 2004), hence the endothelium of vasa vessels is exposed to varying concentrations of oxygen. Thus, the vasa vasorum represents a heterogenous system of vessels/microvessels potentially able to differentially respond, either physiologically or pathophysiologically, to changes in oxygen concentration. Since this microvessel system is damaged/interrupted in, ‘traditional’, CT hSVGs it is prone to pathological sprouting (neovascularization) towards the SV lumenal intima, potentially transporting cholesterol, inflammatory cells, erythrocytes, extracellular matrix and other proatherogenic molecules to the lumen involved in plaque formation and graft occlusion (Sedding et al. 2018). In fact, controversy remains as to the effects that oxygen may have on the vasa vasorum. For instance, it is suggested that stripping of varicose hSV reduces local production of harmful reactive oxygen metabolites (ROM) resulting in a positive effect on vein physiology (Flore et al. 2003). Furthermore, according to Joddar et al. (2015), the higher arterial levels of oxygen and elevated level of reactive oxygen species (ROS) stimulates intimal hyperplasia in hSV organ culture models where the hSVs were harvested by standard endovascular techniques. However, data from such *in vitro* organ culture studies (14 days in the medium, changed every 2 days; some culture medium also containing compound tiron to help reoxygenation) may be limited and may not reflect the true physiological events that occur *in vivo* hSV when used in CABG patients.

A porcine model of SV into artery interposition grafting has been described where the effect of external synthetic stents and sheaths on vein graft remodelling and thickening have been studied (Violaris et al. 1993). It was shown that the placement of a loose-fitting external polyester stent reduced long-term medial and neointimal thickening and platelet derived growth factor (PDGF) expression in this pig model of arteriovenous bypass grafting (Mehta et al. 1998). Here the development and promotion of a neoadventitia in the space between the graft and the stent occurred which allows for the development of microvessels from the native vasa vasorum that “may obviate graft wall hyperplasia through the prevention of graft hypoxia” (Jeremy et al. 2007). In their review on data obtained using this porcine/external stent model the authors state “removal of the saphenous vein, ipso facto, results in a loss of continuity of the vasa vasorum, a microvessel complex that infiltrates and oxygenates large blood vessels which in turn would result in hypoxia of the tissue” continuing, “Disruption of the vasa vasorum, per se, is associated with vascular disease and upregulates the expression of literally hundreds of proteins that include those that promote vein graft disease” (Jeremy et al. 2007). Interestingly, using this porcine model, endothelin-1 and its receptors and nitric oxide synthase – both endothelium-derived factors affected by hypoxia and both affecting vessel growth – are located on vasa vasorum and regions of neovascularization of pig SV grafts (Jeremy et al. 1997, 1998; Wan et al. 2004).

**Fig. 11:**
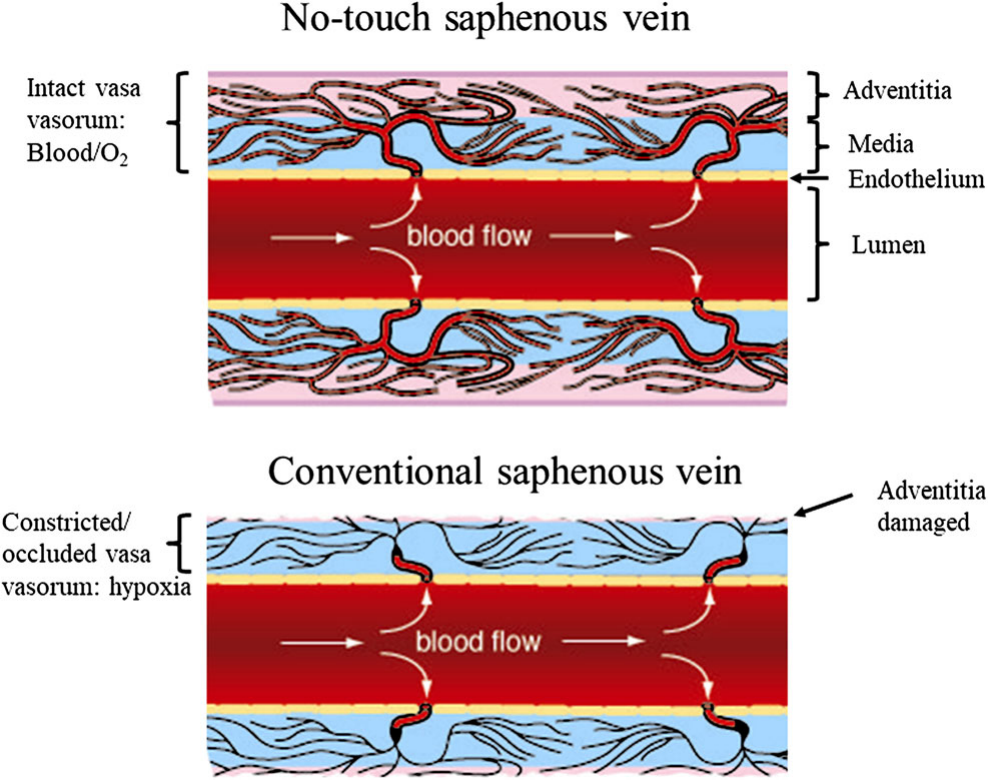
Proposed reduction in blood flow in damaged vasa vasorum: medial hypoxia. **Top panel:** No-touch harvested hSV where the adventitia and vasa vasorum are intact. Blood flow to the vein wall is restored after graft implantation: oxygen and nutrient supply is maintained. **Lower panel:** Diagram of a conventionally harvested hSV where the adventitia has been stripped off or damaged during harvesting. The vasa vasorum constrict or are occluded. Blood flow to the vein wall is reduced releasing hypoxic factors as described in the text. Such factors are associated with many aspects of graft failure including neointimal thickening and atherosclerosis

While a considerable amount of data has been published regarding the role of the vasa vasorum in pathological conditions in human arteries, in particular the epicardial coronary arteries, there is less information available on the SV. Reducing medial blood flow and oxygen supply by occluding the vasa vasorum in arteries causes neointima formation and atherosclerosis via the action of a vast number of hypoxia-induced factors. Since the role of the vasa vasorum in providing oxygen to the wall of the SV is more important than in arteries it seems reasonable to assume that anything reducing blood flow through this microvessel network will produce similar, or more serious, consequences. The considerable damage caused to the vasa vasorum when using CT harvesting described in earlier sections of this review will reduce medial blood flow stimulating a plethora of hypoxia-induced factors involved in vein graft failure (Fig. 11). If the vasa vasorum remains intact, medial blood flow and oxygen supply is maintained and vein graft patency is improved dramatically as shown using the NTSVG (Souza et al. 2006; Samano et al. 2015). According to the recent review by Sedding et al. (2018) “…therapeutic approaches specifically targeting the expanding microvessels in developing plaques will have to be established and evaluated”. Rather than ‘expanding microvessels’ it would seem more logical to harvest the hSV in CABG in a way that preserves an intact, functioning vasa vasorum where no therapeutic intervention would be required.

## Conclusions

The vasa vasorum of the hSV plays a more important role in providing oxygen and nutrients to the vessel wall than in arterial bypass grafts, the ITA and RA. Consequently, this microvascular network is more prolific in the SV than the ITA or RA and extends deeper in the media with some evidence of terminations in the lumen. Furthermore, the connection of vasa vasorum to capillaries within the PVAT surrounding the hSV suggests that, apart from supplying blood, this microvascular network may potentially transport tissue- and/or cell-derived factors from the outermost to the innermost layers of this blood vessel or vice versa. The damage caused to the vasa vasorum of the hSV during conventional harvesting in CABG reduces transmural blood supply, a situation that promotes neointimal hyperplasia and atheroma formation, features associated with mid- and long-term graft patency. If the hSV is harvested with minimal vascular trauma, the vasa vasorum remains intact and arterial blood flow is restored at completion of graft insertion. In addition, an intact vasa vasorum may provide a system for the transport of factors and cell to cell communication across the hSV graft wall that are beneficial to improved graft performance.

## Abbreviations

ANGPT-2: Angiopoietin 2
CABG: Coronary artery bypass graft surgery
CT: Conventional method of harvesting
EVH: Endoscopic vein harvesting
FGF-2: Fibroblast growth factor 2
HIF-1: Hypoxia-inducible factor 1
hNTSVG: Human no-touch saphenous vein graft
hSV: Human saphenous vein
hSVG: Human saphenous vein graft
ITA: Internal thoracic artery
NO: nitric oxide
NOS: Nitric oxide synthase
NT: No-touch method of harvesting
NTSVG: No-touch saphenous vein graft
PDGF-B: Platelet-derived growth factor B
PLGF: Placental growth factor
PVAT: Perivascular adipose tissue
PVF: Perivascular fat
RA: Radial artery
ROM: Reactive oxygen metabolites
ROS: Reactive oxygen species
SCF: Stem cell factor
SDF-1: Stromal cell-derived factor 1
SV: Saphenous vein
VEGF: Vascular endothelial growth factor
VSMC: Vascular smooth muscle cell
